# Effect of Electrode Belt and Body Positions on Regional Pulmonary Ventilation- and Perfusion-Related Impedance Changes Measured by Electric Impedance Tomography

**DOI:** 10.1371/journal.pone.0155913

**Published:** 2016-06-02

**Authors:** Elin Ericsson, Erik Tesselaar, Folke Sjöberg

**Affiliations:** 1 Department of Clinical and Experimental Medicine, Linköping University, Linköping, Sweden; 2 Department of Radiation Physics, Department of Medical and Health Sciences, Linköping University, Linköping, Sweden; 3 Department of Hand and Plastic Surgery and the Burn Clinic, Linköping University, Linköping, Sweden; University of Bari, ITALY

## Abstract

Ventilator-induced or ventilator-associated lung injury (VILI/VALI) is common and there is an increasing demand for a tool that can optimize ventilator settings. Electrical impedance tomography (EIT) can detect changes in impedance caused by pulmonary ventilation and perfusion, but the effect of changes in the position of the body and in the placing of the electrode belt on the impedance signal have not to our knowledge been thoroughly evaluated. We therefore studied ventilation-related and perfusion-related changes in impedance during spontaneous breathing in 10 healthy subjects in five different body positions and with the electrode belt placed at three different thoracic positions using a 32-electrode EIT system. We found differences between regions of interest that could be attributed to changes in the position of the body, and differences in impedance amplitudes when the position of the electrode belt was changed. Ventilation-related changes in impedance could therefore be related to changes in the position of both the body and the electrode belt. Perfusion-related changes in impedance were probably related to the interference of major vessels. While these findings give us some insight into the sources of variation in impedance signals as a result of changes in the positions of both the body and the electrode belt, further studies on the origin of the perfusion-related impedance signal are needed to improve EIT further as a tool for the monitoring of pulmonary ventilation and perfusion.

## Introduction

Ventilator-induced or ventilator-associated lung injuries (VILI/VALI) contribute to increased morbidity and mortality, and the choice of optimal ventilator settings for each individual patient to avoid associated complications is a constant challenge in critical care. Therefore, there is a demand for a bedside technique that can help physicians to optimize the settings [1–3]. During the past 30 years, Electrical Impedance Tomography (EIT) has been developed as a non-invasive, radiation free, bedside monitoring technique for assessing regional distribution of pulmonary ventilation and perfusion [4–8].

EIT of the lungs is based on the application of small alternating currents through a set of electrodes that are positioned in a transverse plane around the thorax. The raw data consists of a series of measured electric potentials that can be converted to impedances and reconstructed as a tomogram, which shows a cross-sectional thoracic image [9]. The impedance of the lungs changes continuously as a result of both ventilation and perfusion. While ventilation-related changes in impedance are caused by regional differences in air volume, the exact mechanisms behind the perfusion-related changes in impedance remain less clear [10].

An important and relevant feature of pulmonary ventilation and perfusion when it comes to mechanical ventilation, is that their distributions are not homogeneous. Traditionally, it has been suggested that gravity and the morphology of the lung are the two main factors that contribute to the non-homogeneous distribution [11,12], and so the largest variations are seen along the gravity vector. However, recent studies have shown that both ventilation and perfusion are heterogeneous in the iso-gravitational plane as well. In fact, the variations in ventilation and perfusion have been found to be larger within the same horizontal plane than between different ones [13]. These variations correlated both spatially and temporally and it has therefore been suggested that the heterogeneity is the result of the geometry of the airways and the main vascular structures in the thorax [11].

So far we know of few studies of EIT that have detected regional differences that are in agreement with the findings of modern research on the distribution of pulmonary ventilation and perfusion, i.e. studies that show heterogeneity of ventilation- and perfusion-related impedance changes, both along the gravity vector and within each plane. The fact that most studies that have used EIT have been done with the body in different positions makes direct comparison of results difficult. In a recent study in which the primary aim was to compare different methods of filtering for the perfusion-related impedance signal, the authors found no gravity-dependent effect on the distribution of ventilation-related impedance changes when the patients were supine and prone, respectively [14]. Similar findings were reported in a study that investigated the gravity-related effect on ventilation-related impedance changes at the sixth intercostal space during parabolic flights [15]. Conversely, other studies of EIT have detected distribution patterns of ventilation-related impedance changes, which are attributable to gravity, when subjects were studied in different postures [16,17]. One of these studies also investigated the effect of placing the electrodes in transverse planes at different thoracic levels (fourth and seventh intercostal space, parasternally) and found significant differences in the ventilation-related impedance signal between the levels. The authors explained the results by relating them to the anatomy and physiology of the thorax and the lungs [17].

Clearly, there is disagreement about how ventilation-related and perfusion-related changes in impedance are linked to physiological changes that result from different positions of the body. Moreover, the number of studies on impedance changes related to different thoracic levels are very limited. We hypothesized that if we studied changes in impedance related to ventilation and perfusion using an EIT-system with 32 electrodes, with the electrodes at three different thoracic levels and the body in five different positions, we could provide insights into how regional differences in ventilation and perfusion are reflected in the impedance signals.

The aim of this study was therefore to measure impedance changes related to ventilation and perfusion using a 32-electrode EIT system, and to find out how they are affected by the position of the body and the placing of the electrodes in healthy subjects.

## Subjects and Methods

### Subjects

Ten, healthy men were recruited (mean age (SD), 23 (3) years; mean weight (SD), 78 (10) kg; mean height (SD), 180 (10) cm). Each subject gave written informed consent before the investigation started. The study was approved by the Regional Ethics Review Board at Linköping University, Sweden. All subjects were non-smokers and stated that they had no history or symptoms of pulmonary or cardiac disease. They had been instructed not to have any strenuous exertion for two hours before the investigation. For each subject the three thoracic levels at which the electrode belt was placed were identified by manual palpation in the parasternal line, and marked on the chest wall at the front. The belt was placed in a transverse plane, not following the oblique shape of the costae, and pen markings on the back were added afterwards. The marks were used to make it easy to see if the electrode belt became displaced. The three levels used corresponded to the second (i2), fourth (i4) and sixth (i6) intercostal space.

### Acquisition of data

Data were acquired using the Enlight platform (Experimental Pulmonology Laboratory and Polytechnic Institute of the University of São Paulo, in a partnership with Dixtal Biomédica Ltda., São Paulo, Brazil) [18]. The device is capable of producing 50 images/second of impedance recordings from 32 electrodes positioned equidistantly around the thorax. Small electric currents (5 mA; 125 kHz) are applied through pairs of electrodes in a rotating sequence, with one passive electrode interposed. During a current-application pattern, 29 differential voltages are measured between remaining pairs of electrodes. One complete acquisition cycle of 32 current patterns produces 928 measurements of voltage that comprised one “raw voltage frame” used as input for a”relative EIT image”. These images are generated by a reconstruction algorithm for a cross-section of the thorax, which is based on a sensitivity matrix derived from a three-dimensional finite element model. The images are “relative” because they are created by comparing the most recent raw voltage frame with a reference or baseline frame chosen by the investigator. Output pixel values indicate percentage changes in local tissue impedance, from the reference to the present frame.

An electrode belt with 32 electrodes, connected to the EIT device, was placed around the thorax and one reference electrode on the abdomen. The belt consisted of a flexible rubber band (84 cm long and 1 cm wide) with 32 protruding metal plate electrodes (contact area 11.3 x 7 mm) threaded on to it. The ends of the belt were connected by a hook. Electrode gel (Lectro Derm LD-3, Viroderm AB, Solna, Sweden) was applied between the electrodes and the surface of the skin. Before it was applied the skin was swabbed with chlorhexidine ethanol (5 mg/ml, Fresenius Kabi, Uppsala, Sweden). To prevent the belt from sliding out of place, we created braces out of two elastic bandages. Depending on which electrode belt position was being investigated, the lengths of the braces were adjusted accordingly.

### Protocol

For each electrode belt position (i2; i4; i6), measurements were made with the body in five different positions: standing, supine, prone, left lateral, and right lateral. The order in which the positions of the electrode belt and the positions of the body were studied, was randomized. In each position the subjects were instructed to breathe in three different ways, which they had practiced beforehand (Table 1). This included breath holding at functional residual capacity + tidal volume (FRC + V_T_) for 20 seconds for assessment of perfusion-related impedance changes. The position of the electrodes and each electrode contact area were inspected regularly and corrected when necessary. All measurements were made in a temperature-controlled room.

**Table 1:** Description of the breathing maneuvers during electrical impedance tomography.

Breathing maneuver		Duration	Purpose
1	Spontaneous tidal breathing	3 minutes	Stabilize breathing, acquire ventilation-related impedance signal
2	Breath holding at FRC + V_T_	20 seconds	Acquire perfusion-related impedance signal
3	Spontaneous tidal breathing	1 minute	Recover from breath hold maneuver

FRC = functional residual capacity; V_T_ = tidal volume.

### Analysis of EIT data

The reference image was defined by taking the mean of the first 6 seconds of data acquired at each electrode belt position and in each position of the body. Data obtained from the EIT software were analysed using custom-built algorithms in Matlab R2007b (The Mathworks Inc., Natick, MA). For each of the 32 x 32 pixels in each image, a plethysmograph was constructed that consisted of the change in impedance in that pixel over time. These plethysmographs were then analysed by “ensemble averaging”. For each pixel, a representative impedance waveform was constructed, either by taking the mean of about 12 breathing cycles (the last minute of three during tidal breathing), or about 20 heartbeats (during the breath holding maneuver), for ventilation and perfusion, respectively [19]. This analysis was made for each electrode belt position and position of the body. Measures for ventilation and perfusion were defined as the amplitude of the representative impedance waveforms. The ventilation-related changes in impedance were calculated as the decrease from the peak impedance value seen during inspiration to the smallest impedance value seen during expiration. Similarly, the perfusion-related impedance changes were calculated as the decrease from the peak impedance value seen during diastole to the smallest impedance value seen during systole. This procedure results in an automatic exclusion of the ventricles, as the impedance curve of the ventricles is inverted to the perfusion-related impedance curve, and negative values were set to zero [6,20–22].

The images were then divided into four equally-sized, 16x16-pixel regions of interest (ROI): anterior, posterior, right, and left. For each ROI, the sums of the impedance amplitudes in each pixel (area under the curve) were calculated and denoted as ΔZ_V_ (ventilation-related change in impedance) and ΔZ_Q_ (perfusion-related change in impedance) respectively. Consequently, eight different variables are mentioned below, for example ΔZ_V, right_ indicating the ΔZ-value for ventilation in the right ROI and ΔZ_Q, anterior_ indicating the ΔZ-value for perfusion in the anterior ROI.

### Statistical analysis

For statistical analysis we used the software Prism, version 5.04 for Windows (GraphPad Software, San Diego, CA, USA) and Microsoft Office Excel 2003. Comparisons of ROI within one position of the body were made using paired Student’s *t* tests. Assessments of possible differences in distribution between different positions of the body were made by calculating the ratio between the ΔZ_V, right_ or ΔZ_Q, right_ and the total ΔZ_V_ or ΔZ_Q_ (right + left lung). The mean ratios were then analysed using paired Student’s *t* tests.

The comparisons of ΔZ_V_ between i2, i4, and i6 were made by comparing the sum of all pixels in a cross-section using the repeated measures one-way ANOVA together with a post hoc test for linear trend. Comparisons of ΔZ_Q_ between i2, i4, and i6 were analyzed with the same method but with Bonferroni’s test. Bonferroni’s test was chosen over Tukey's test because it is more conservative and reduces the risk of a type II error.

Comparisons of amplitude within i2, i4 and i6 were also made between different positions of the body using repeated measures one-way ANOVA with Bonferroni’s test. Probabilities of less than 0.05 were accepted as significant.

## Results

Anthropometric data for the subjects participating in the study are given in Table 2. The mean (SD) temperature in the room was 23.1 (0.3°C during the measurements.

**Table 2:** Anthropometric data in 10 subjects.

Variable	Mean (SD) (n = 10)
Age (years)	23 (3)
Height (cm)	180 (10)
Weight (kg)	78 (10)
Body mass index (kg/m^2^)	24 (2)
Blood pressure (mmHg)	
Systolic	124 (9)
Diastolic	73 (6)
Chest circumference (cm)	
i2	99 (7)
i4	97 (9)
i6	91 (9)

The second, fourth and sixth intercostal space is denoted i2, i4 and i6 respectively.

### Distribution of ventilation-related impedance changes

**Fig 1:**
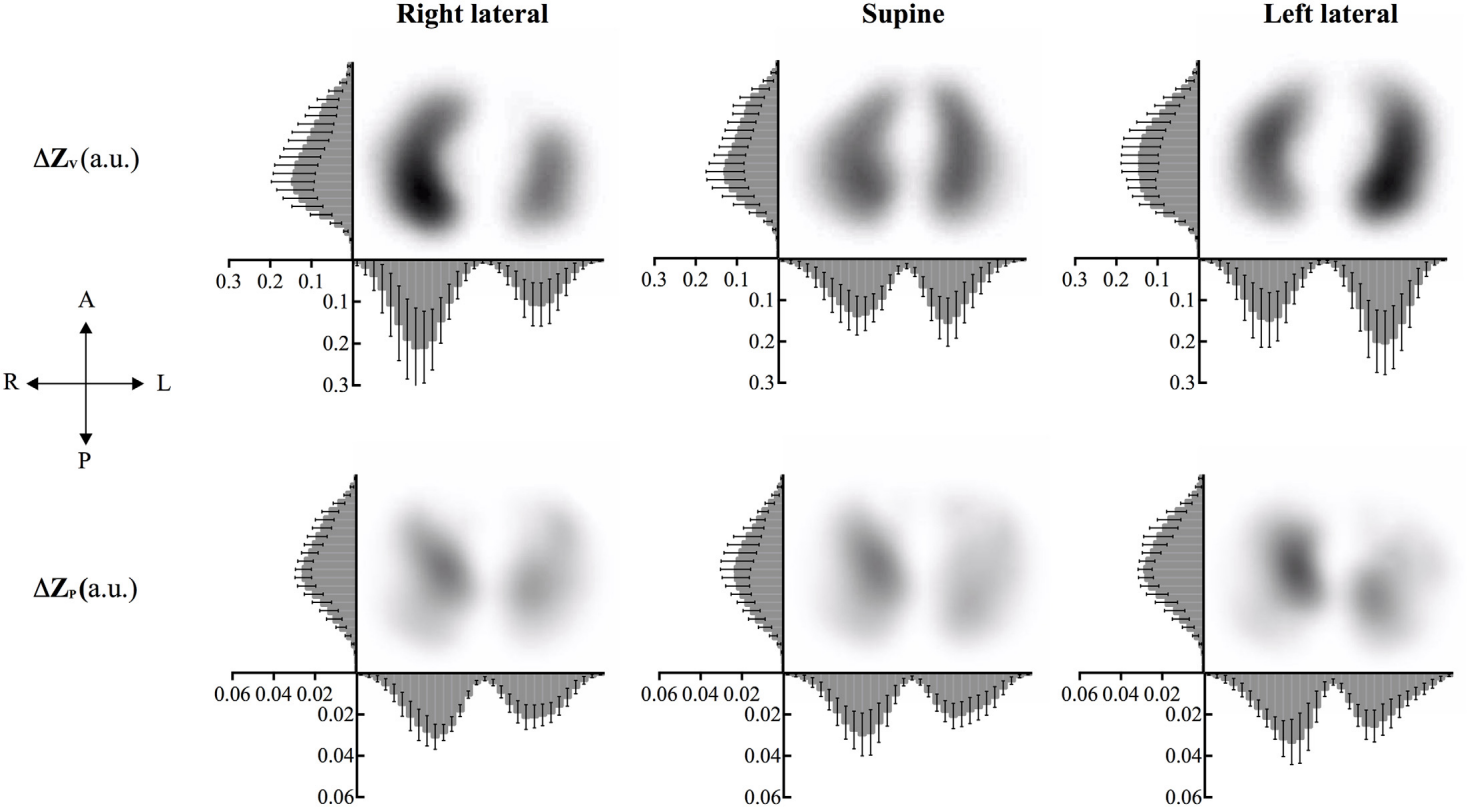
Typical distribution of ventilation-related and perfusion-related changes in impedance during tidal breathing with the subject supine, and in right and left lateral positions as measured with EIT at the 6th intercostal space. The distribution is presented in the anteroposterior and right-left axis, respectively. Each data point indicates the mean (SD) of respective impedance amplitude (arbitrary unit) for 10 subjects. Note that the right side of the images corresponds to the left lung and vice versa. A complete analysis of the distributions of the changes in impedance for all electrode belt and body positions is presented as supplemental material.

Fig 1 shows a typical example of the distribution of the ventilation-related and perfusion-related changes in impedance in the anteroposterior axis and the right-left axis, respectively, for the supine, right, and left lateral positions at the level of i6. Images showing the distributions of ventilation-related impedance changes and perfusion-related impedance changes are given in S1 and S2 Figs, respectively. Here, only a numeric summary of the most important findings will be presented (Table 3). When standing, as far as the anterior-posterior distribution was concerned, there were larger values for ΔZ_V_ posteriorly, but this was significant only in i6. When the subject was supine, ΔZ_V_ was larger in the anterior ROI than in the posterior ROI at all electrode belt positions, but the difference was significant only at i2. Also, ΔZ_V, right_ was higher than ΔZ_V, left_ at the level of, i4 and i6 (i4: *p* = 0.012; i6: *p* = 0.013). Also, with standing and prone positions, ΔZ_V, right_ was significantly higher than ΔZ_V, left_.

**Table 3:** Comparisons of the right-left distributions and the anterior-posterior distributions of ventilation-related impedance changes between body positions.

Position of body and electrode belt	Right lung (% of total)	Posterior lung (% of total)
Standing		
i2	**54.6**	50.2
i4	**54.1**	54.1
i6	**56.5**	**54.9**
Supine		
i2	51.3*	39.5***
i4	**52.8**	48.0*
i6	52.7*	48.3**
Prone		
i2	**53.2**	49.4†††
i4	57.1*††	51.2
i6	**57.1**††	54.2†††
Right lateral		
i2	68.0***†††	45.9*†
i4	74.3***†††	51.3†
i6	67.4*†	**53.7**†
Left lateral		
i2	41.6***†	44.9†
i4	38.3**††	48.2
i6	42.6*	50.9

The numbers represent means of percentages of ventilation-related impedance changes that are in the right and posterior lung. Boldfaced numbers indicate a significant difference between left and right, or anterior and posterior.

*p < 0.05;

**p < 0.01;

***p < 0.001 compared with standing position for the same position of the electrode belt.

^†^*p* < 0.05;

^††^*p* < 0.01;

^†††^*p* < 0.001 compared with supine position for the same position of the electrode belt.

There were significantly higher values for ΔZ_V_ in the dependent lung in the right lateral and left lateral positions for all electrode belt positions, except for the left lateral position in i6 (*p* = 0.18).

### Distribution of perfusion-related impedance changes

The right-left distributions and the anterior-posterior distributions of perfusion-related impedance changes for the different positions of the body and the electrode belt are summarized in Table 4. When subjects were standing, supine, and prone, ΔZ_Q_ was larger in the anterior ROI than in the posterior ROI at the level of i4 and i6. At the level of i2, there was no longer a difference. During standing ΔZ_Q, right_ was significantly larger than ΔZ_Q, left_ at the level of i2. When the subject was supine, ΔZ_Q_ was significantly higher in the right lung compared with the left lung at i6. When the subject was prone, ΔZ_Q, right_ was larger than ΔZ_Q, left_ at the level of i4 and i6. In the right lateral position, ΔZ_Q_ was higher in the dependent lung for all three electrode belt positions whereas in the left lateral position, ΔZ_Q_ was higher in the non-dependent lung but this was only significant in i2.

**Table 4:** Comparisons of the right-left distributions and the anterior-posterior distributions of perfusion-related impedance changes between body positions.

Position of body and electrode belt	Right lung (% of total)	Posterior lung (% of total)
Standing		
i2	**53.7**	46.7
i4	47.9	**35.8**
i6	44.3	**35.7**
Supine		
i2	51.5	49.1
i4	48.1	**46.3**
i6	**56.4**	**46.0**
Prone		
i2	52.6	48.0
i4	**55.9**	**41.2**
i6	**56.1**	**35.3**
Right lateral		
i2	**54.9**	50.3
i4	**55.3**	**44.3**
i6	**57.0**	**41.9**
Left lateral		
i2	**55.2**	48.9
i4	54.0	**44.5**
i6	55.3	**40.8**

The numbers represent means of percentages of perfusion-related impedance changes that are in the right and posterior lung. Boldfaced numbers indicate a significant difference between left and right, or anterior and posterior.

### Effect of the position of the electrode belt on impedance changes

**Fig 2:**
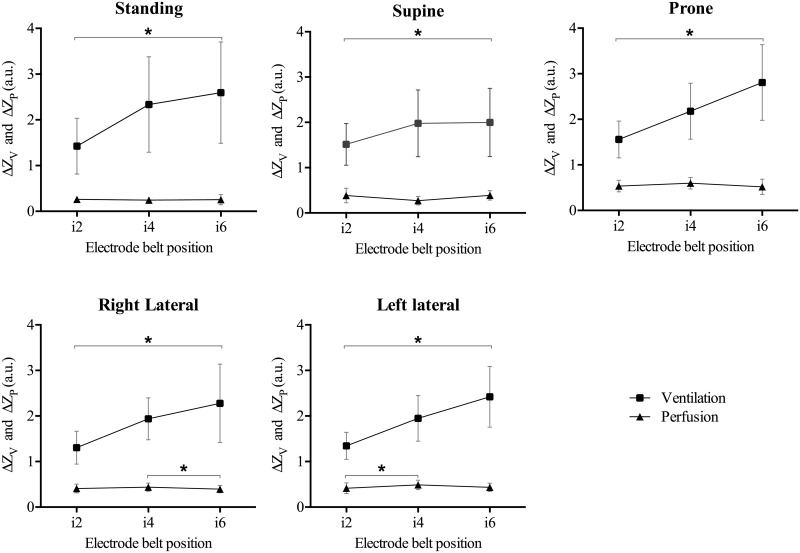
Comparison of mean ventilation- and perfusion-related impedance amplitude values, respectively, between the different electrode belt positions for each position of the body. Exact numbers can be found in Table 4. * indicates *p* < 0.05.

There was a significant increase in the total ventilation-related impedance changes (ΔZ_V, left_ + ΔZ_V, right_) from i2 to i6, (as tested using post hoc test for linear trend) which indicated that ventilation was increased from i2 to i6 in all five positions (all *p* < 0.001, Fig 2).

In contrast to ventilation, there was no such trend in total perfusion-related changes in impedance (ΔZ_Q, left_ + ΔZ_Q, right_) between the different belt positions, although there were some significant differences between belt positions for the lateral body positions (Standing: *p* = 0.87; Supine: *p* = 0.08; Prone: *p* = 0.06; Right lateral: *p* = 0.02; Left lateral: *p* = 0.006) (Fig 2).

### Effect of the position of the body on impedance changes

**Fig 3:**
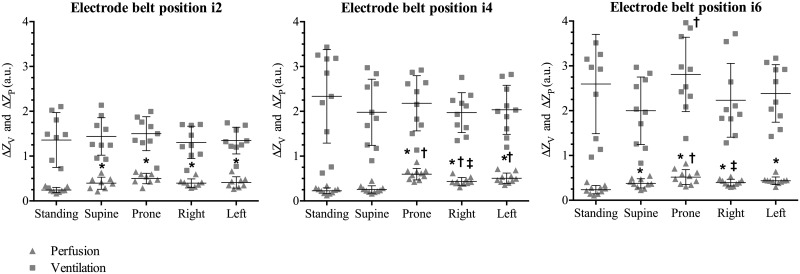
Comparison of mean impedance amplitude values for ventilation and perfusion, respectively, among the different positions of the electrode belt. Markers indicate data points from each subject. * denotes significant difference compared with standing position; † denotes significant different compared with supine; ‡ denotes significant difference compared with prone.

The ventilation-related changes in impedance ΔZ_V_ did not change among positions of the body within the same electrode belt position, (Fig 3) except for prone in i6, where ΔZ_V_ was significantly larger than when supine (*p* = 0.02).

Considerably more variation in the perfusion-related changes in impedance were found between different body positions. At i2, ΔZ_Q_ was significantly larger in all the horizontal positions than when standing (Supine: *p* = 0.002; Prone: *p* < 0.001; Right lateral: *p* = 0.002; Left lateral: *p* < 0.001). At i4, there was a significantly larger ΔZ_Q_ in all horizontal positions except supine when compared with standing (all horizontal positions *p* < 0.001). ΔZ_Q_ was significantly larger when subjects were prone than when they were supine or in the right lateral positions both at i4 and i6. At i4, ΔZ_Q_ was smaller when subjects were supine than in the right and left lateral positions. Lastly, at the level of i6, ΔZ_Q_ in standing subjects was significantly smaller than when the subject was horizontal (Supine: *p* = 0.008; Prone: *p* < 0.001; Right lateral: *p* = 0.002; Left lateral: *p* < 0.001).

## Discussion

Few studies have examined the ability of EIT to investigate how regional differences in ventilation and perfusion change with varying positions of the body and the electrode belt [5,8,15–17]. This study contains partly new and more extensive research. We chose the standing over the sitting position because it allows gravity to work freely without any disturbance.

### Distribution of ventilation-related impedance changes

When the subject was standing the observed distribution of ventilation (as measured using ventilation-related changes in impedance) was as expected. Dominant ventilation in the right lung can be explained by the fact that the right lung is larger than the left, and this difference becomes more distinguishable towards the bottom of the craniocaudal axis. The larger changes in ventilation-related impedance in the dorsal regions of the lungs also confirm the traditional views on normal anatomy, with more lung tissue in the posterior regions than in the anterior regions [23]. When the subject was supine the ventilation was more evenly distributed between the left and right lung, compared with when standing, although the right lung was still considerably more ventilated. We speculate that this is caused by a shift in the position of the abdominal organs, particularly the liver, which exerts greater pressure against the diaphragm and the organs of the thorax when there is a change from standing to supine [24,25].

The findings of the distribution of ventilation-related impedance changes when the subject was supine and prone contrasted with the gravitational model describing distribution of ventilation. We found a significant difference between the dependent and the non-dependent regions in only 2/6 combinations of electrode placement and position of the body. In these cases there was more ventilation in the non-dependent part of the lungs. Interestingly, the distribution of ventilation between the non-dependent and dependent regions in these positions have been contradictory in previous studies [5,16,17]. Frerichs et al. investigated the dependence of pulmonary ventilation on gravity in five healthy subjects at the sixth intercostal space. Spontaneous tidal breathing was measured in five different positions: sitting, supine, prone, and right and left lateral. The results were consistent with the gravitational model and presented a distinct redistribution of ventilation in favor of the dependent regions [16]. Conversely, a recent study by Grant et al. found no gravity-dependent effect on the distribution of ventilation when subjects were supine and prone [14]. Reifferscheid et al. investigated subjects sitting, supine, and in the right lateral position, and suggested that there was more ventilation in the anterior region at i4 and in the posterior region at the seventh intercostal space [17].

In the present study we found a relatively homogeneous distribution of ventilation-related impedance changes in the anteroposterior axis, except for the significantly larger changes that we found in the posterior lung in prone subjects at the level of i6. This can be explained by a more homogeneous distribution of the transpulmonary pressure resulting in less distension of the alveoli in the non-dependent regions and more homogeneous alveolar inflation. This method has been used to improve oxygenation in patients with acute respiratory distress syndrome for several years [26,27].

In contrast to supine and prone, there were significantly greater changes in ventilation-related impedance in the dependent lung at i4 and i6 in the lateral positions, which confirms previous findings [8,14,16,17]. The lack of difference between the left and right lung in the left lateral position at the level of i6 could indicate a combined effect of compression by the heart on the basal lung tissue and the greater amount of lung tissue in the right hemithorax. In addition, there is a possibility that the right hemithorax is less affected by the compressing effect of the liver when the body is in the left lateral position. The plane at the level of i2 is the most cranial of the measurements that can be made with EIT. Nevertheless, a considerable amount of lung tissue is still present above i2, and we conclude that there is enough to show significant differences between the right and left lungs.

The fact that there is a clear redistribution of tidal volume in favor of the dependent lung in both lateral positions, but no clear difference between the dependent regions when supine or prone, confirms the explanation provided by Grant et al. They suggested that gravity contributes to the distribution of ventilation through its effect on the anatomy of the lungs [14], as shown by the fact that a switch from supine to prone or vice versa has little effect on the positions of the organs inside the chest. Changes between lateral positions, however, result in more movement of lung tissue, hence the significant differences between these positions. Also, gravity affects the curvature of the diaphragm when in lateral position, due to the shift of abdominal content, which also affects ventilation when in the lateral position. Finally, the gravity-induced deformation of the lungs is also a significant factor in the change of distribution of the transpulmonary pressure when moving between supine and prone [26,27].

### Distribution of perfusion-related impedance changes

In contrast to the results of the distribution of ventilation-related impedance changes, the results of the distribution of perfusion-related impedance changes contradicted the theory of a gravity-dependent distribution of pulmonary perfusion. There were significantly larger perfusion-related impedance changes in the anterior lung than in the posterior lung in i4 and i6 for all five positions. These results were partly unexpected, as we thought that the larger amount of lung tissue dorsally would be apparent. However, the cardiac-related impedance changes associated with pulmonary perfusion also originate from the atria and the large vessels inside the thorax. Together with the right and left atria, vessels such as the aorta, superior and inferior vena cavae, and the pulmonary arteries, have reduced impedance during systole [21,22] and can therefore influence the distribution of perfusion-related impedance measured with EIT. The dominance of perfusion in the anterior lung in all positions of the body that we found could be explained by the relatively anterior position of larger vessels and the atria.

Significantly larger, perfusion-related changes in impedance were seen in the dependent lung in the right lateral position at all three electrode belt positions compared to the non-dependent lung. This could be the result of the combined effects of the larger right lung, the effect of gravity on the lung tissue [12], the right atrium and the superior and inferior venae cavae that are running through the right part of the thorax. The greater changes in perfusion-related impedance in the non-dependent lung in the left lateral position at i2 might be the result of the combined perfusion seen in the lung tissue and the superior vena cavae [23]. It is also possible that regional variations in hypoxic vasoconstriction contribute to the variations in perfusion-related impedance [28]. At i4 and i6 this difference was no longer significant and could be attributed to the fact that the descending aorta runs through the left side of the thorax, and a possible leftward shift of the right atrium.

We know of only one previous study on the distribution of perfusion related to changes in position, and that found no gravity-related differences in perfusion in the four positions investigated (supine, prone, and right and left lateral) [14].

### Position of the electrode belt

The increasing changes in ventilation-related impedance along the craniocaudal axis implies that the ventilation is less in the apices, and greater in the bottom of the lungs, which confirms the traditional view of how the air is distributed throughout the lungs during tidal breathing [11–13]. However, the difference between the three electrode belt positions and the observed linear trend does not entirely disappear when the subjects moved from standing to supine. This further suggests that gravity is one of only several factors that affect the distribution of ventilation.

In contrast to the ventilation-related impedance changes, no such linear trend was found between the electrode belt positions for the perfusion-related signal. We found no differences between the three electrode placements whether the subject was standing, supine, or prone. The theory of gravity-dependent distribution made this unexpected [11,13], and the increasing amount of lung tissue along the craniocaudal axis should perhaps have been apparent in these data. The larger impedance changes in i4 compared with i6 in the right lateral position could result from the compressing effect of the liver [24,25], while the larger impedance changes in i4 compared with i2 in the left lateral position might reflect the greater amount of lung tissue in i4 and the leftward shift of the atria.

The total ventilation-related change in impedance at a given electrode belt position did not vary much among positions of the body. The overall perfusion-related change in impedance, however, was more variable. The larger perfusion-related signal in i2 when the subject shifted from standing to a horizontal position is what would be expected if the distribution of perfusion is dependent on gravity. The fact that this difference disappeared at i4 could be explained by the compressing effect of the heart [29]. This explanation is also supported by the fact that at i6, all four horizontal positions showed stronger perfusion-related signals than when standing. The relatively high values of perfusion-related impedance changes when subjects were prone compared with supine in i4 and i6 could be explained by smaller compressive effect of the heart [29] and of the abdominal organs, respectively. Finally, the compressive effect of the liver could be why the perfusion-related signals in the right lateral position were smaller than those at i6 when the subject was prone [24].

These results together suggest that the distribution of ventilation-related impedance changes among the electrode belt positions is relatively independent of how the body is positioned, but that the distribution of perfusion-related impedance changes varies considerably between positions, presumably as a result of gravity or compression of the lung by other organs.

### Limitations

We acknowledge that we have used no direct reference method to validate the distribution of ventilation and perfusion. The validation of how exactly the acquired signals in EIT indicate pulmonary ventilation and perfusion is challenging, as it requires advanced imaging methods that often involve ionizing radiation. Consequently, the validation studies on EIT with these methods have been made on animals or patients who were already listed for imaging [4,5,7,30–33]. Particularly the interpretation of impedance changes related to the cardiac-cycle, commonly described as “perfusion-related”, has been debated [9,10,21]. Because these changes in impedance arise from cyclic changes in volume of blood in the lungs, heart and major vessels, they do not strictly measure perfusion in the definition of forward blood flow. Alternative definitions including “circulation-dependent impedance changes” and “pulsatility” have therefore been suggested and could be more appropriate. Because the perfusion data were extracted from a 20-second period of apnea it is possible that important interactions with changes in ventilation are missing. However, a previous study showed that this was not the case [14]. Although the electrode belt was firmly secured level with i2 with braces, and pen marks on the front and back of the subjects, there was some displacement. This happened most often below the armpits where the belt tended to slide down 1–2 cm. Concerns about whether the use of the belt had any effect on thoracic compliance or not, depending on the tightness of the belt, have also previously been raised [34]. We used an elastic belt with a fixed length and although the inclusion criteria stated men of normal weight, there were still distinct differences in chest circumference. However, we did not investigate whether this could have affected the results. The measurements at the different electrode levels were not made simultaneously and may therefore have been registered during different circumstances. Lastly, a recent study found that a 15-minute stabilisation period after any change in body position was needed in order to correctly asses regional distribution of ventilation using EIT [35]. In our study, the acquiring of data always started as soon as the subjects had changed their body position and after the electrodes and signal had been checked.

## Conclusions

EIT enabled us to detect regional changes in distribution of ventilation that arose with the body in different positions and with different electrode belt positions. The ventilation-related changes in impedance along the craniocaudal axis were in agreement with traditional respiratory physiology. The distribution of the perfusion-related changes in impedance seemed to be less affected by gravity and followed rather irregular patterns, which may be explained by interference of the larger vessels inside the thorax, which are likely to contribute to the impedance signals. Further attempts to understand the origin of the perfusion signal are needed and investigations about whether spatial resolution may make it possible to filter out the large vessels of the thorax are also necessary.

## Supporting Information

S1 Dataset (ZIP)

S1 Fig Distribution of ventilation-related impedance changes for different positions of the body and the electrode belt. (PDF)

S2 Fig Distribution of perfusion-related impedance changes for different positions of the body and the electrode belt. (PDF)
