# Supplementary material for: Effect of Electrode Belt and Body Positions on Regional Pulmonary Ventilation- and Perfusion-Related Impedance Changes Measured by Electric Impedance Tomography
Source: PLoS One. 2016 Jun 2;11(6):e0155913. doi: 10.1371/journal.pone.0155913 (PMC4890811; doi:10.1371/journal.pone.0155913)
Supplement: S2 Fig — (PDF) [file pone.0155913.s003.pdf]

S2 Figure.

A. Distribution of perfusion-related impedance changes for the supine, right, and left lateral positions and for each electrode belt position.

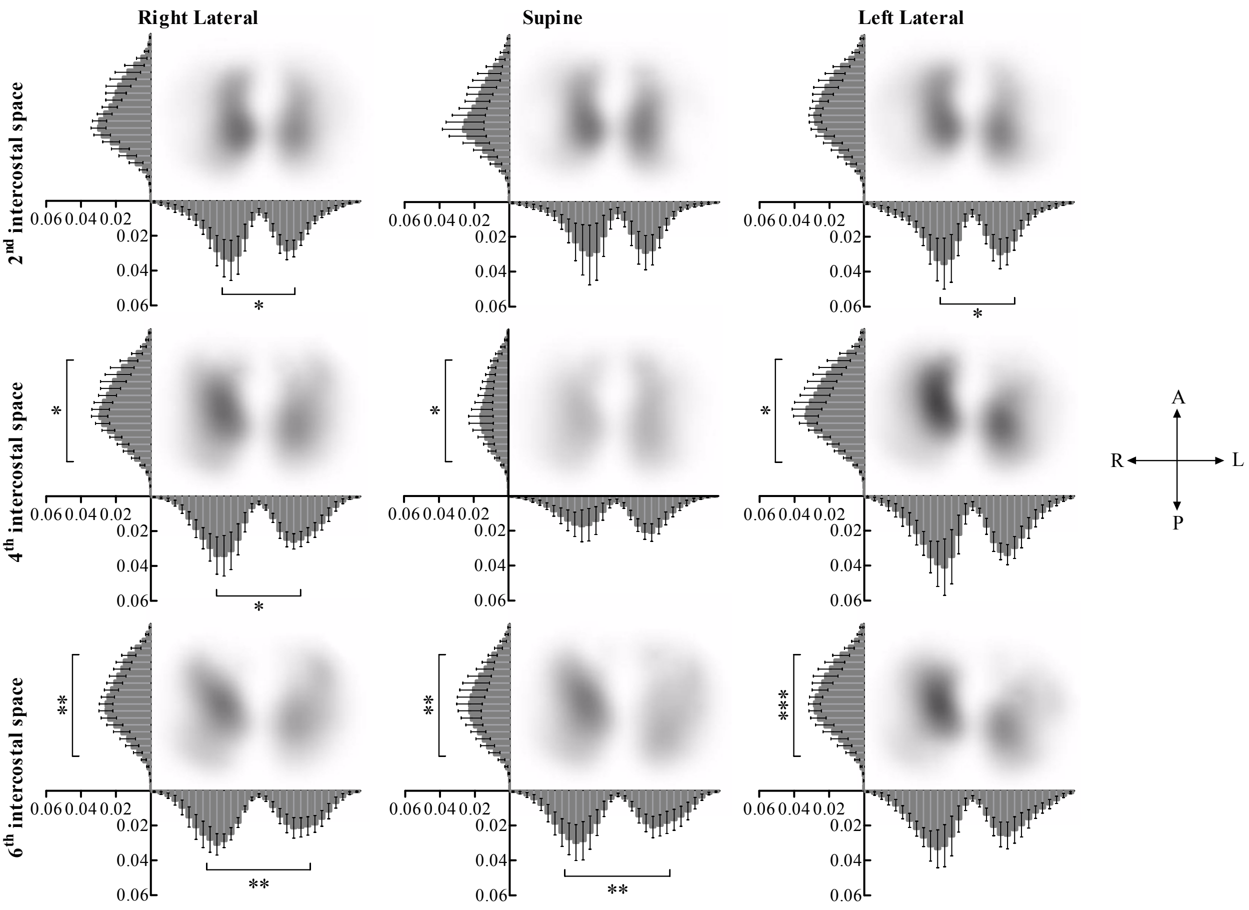

Perfusion-related impedance changes in the supine, and right and left lateral positions for each electrode belt position during breath-holding at FRC+VT for about 20 seconds. The distribution is presented in the anteroposterior and right-left axis, respectively. Each data point indicates the mean (SD) of respective impedance amplitude (arbitrary unit) for 10 subjects. *p < 0.05; **p < 0.01; ***p < 0.001 denote significant differences between the dependent and independent regions of interest (ROI) (anteroposterior and right-left, respectively). Note that the right side of the images corresponds to the left lung and vice versa.

B. Distribution of perfusion-related impedance changes for the standing, supine, and prone positions and for each electrode belt position.

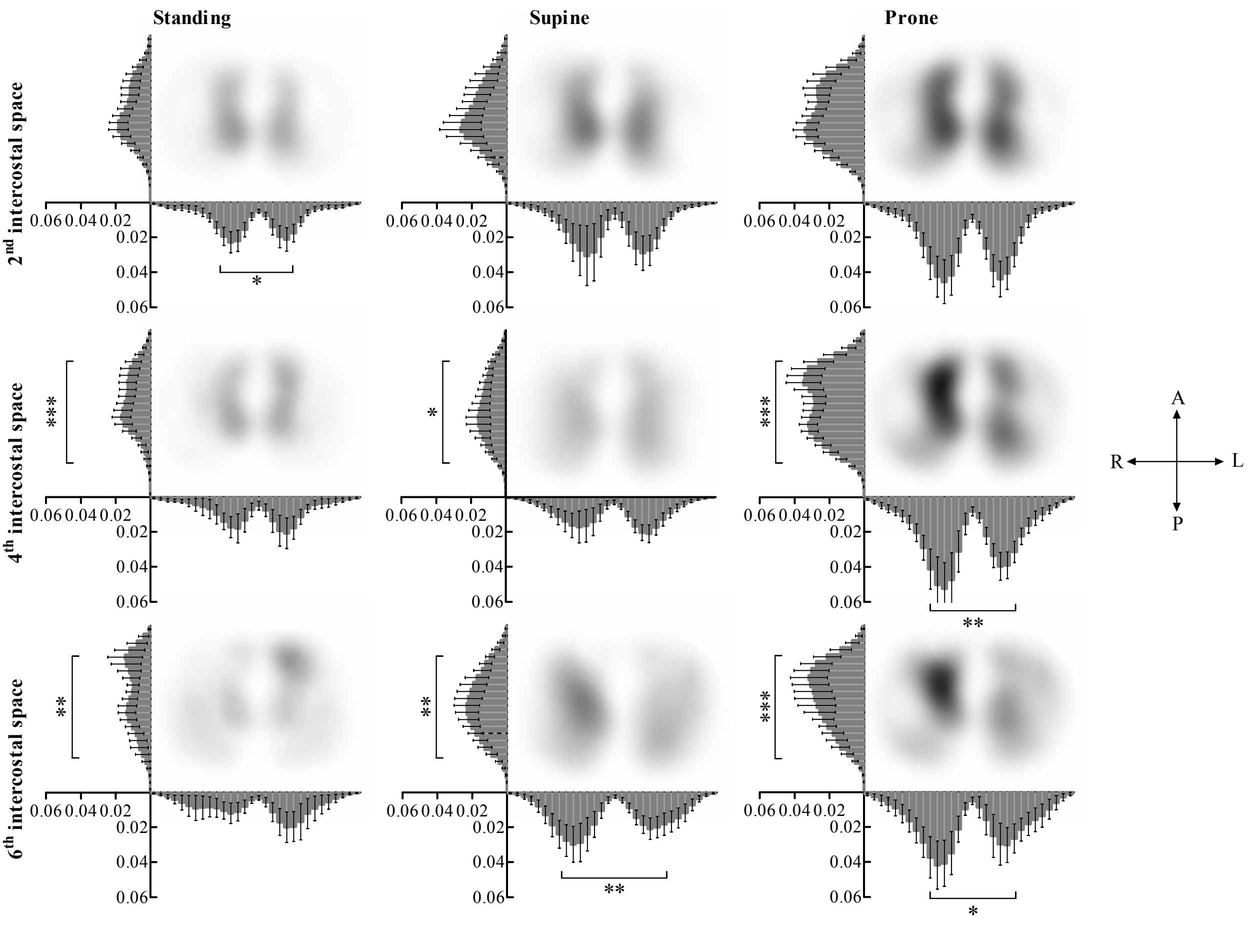

Perfusion-related impedance changes with the subjects standing, supine, and prone for each electrode belt position during breath holding at FRC+VT for about 20 seconds. The distribution is presented in the anteroposterior and right-left axis, respectively. Each data point indicates mean (SD) of respective impedance amplitude (arbitrary unit) for 10 subjects. *$p < 0.05$; **$p < 0.01$; ***$p < 0.001$ denote significant differences between the dependent and independent regions of interest (ROI) (anteroposterior and right-left, respectively). Note that the right side of the images corresponds to the left lung and vice versa.
